# ﻿Ten new species of genus *Tachycines* (Orthoptera, Rhaphidophoridae, Aemodogryllinae) from karst caves in Guizhou, China

**DOI:** 10.3897/zookeys.1109.73937

**Published:** 2022-07-01

**Authors:** Xulin Zhou, Weicheng Yang

**Affiliations:** 1 School of Life Sciences, Guizhou Normal University; Guiyang, Guizhou 550031, China; 2 Guizhou Institute of Mountain Resources, Guiyang, 550001, China; 3 Institute of Karst Caves, Guizhou Normal University, Guiyang, Guizhou 550031, China

**Keywords:** Aemodogryllini, caves, Guizhou, *Gymnaeta*, new species, rhaphidophorids

## Abstract

Ten new karst cave-dwelling rhaphidophorids species of the subgenus Gymnaeta of the genus *Tachycines* are described from Guizhou Province, southern China; i.e., Tachycines (Gymnaeta) zhongi**sp. nov.**, Tachycines (Gymnaeta) jinniui**sp. nov.**, Tachycines (Gymnaeta) shibenzhangi**sp. nov.**, Tachycines (Gymnaeta) lahaidensis**sp. nov.**, Tachycines (Gymnaeta) pinglangus**sp. nov.**, Tachycines (Gymnaeta) shanduensis**sp. nov.**, Tachycines (Gymnaeta) buyii**sp. nov.**, Tachycines (Gymnaeta) portae**sp. nov.**, Tachycines (Gymnaeta) ziyunensis**sp.nov.**, and Tachycines (Gymnaeta) jialiangensis**sp. nov.** All specimens were collected from Guizhou Plateau.

## ﻿Introduction

The subgenus Gymnaeta Adelung, 1902 belongs to the tribe Aemodogryllini that is comprised of 67 species predominantly distributed in China, with eight species extending southwards to Southeast Asia: six in Vietnam, one in Myanmar, and one in the Philippines (Adelung 1902; Karny 1926, 1929, 1934a, b; Gorochov 1994, 1998, 2001, 2010, 2012; Zhang and Liu 2009; Qin et al. 2019; Cigliano et al. 2021). In addition to the surface species, several species of the subgenus Tachycines (Gymnaeta) inhabit cave habitats (Gorochov 2001; Gorochov et al. 2006; Jiao et al. 2008; Rampini et al. 2008; Feng et al. 2019, 2020; Qin et al. 2019; Zhou and Yang 2020; Zhu et al. 2020).

In recent years, the interest in cave organisms research has increased, and many cave beetles, spiders, cave crickets, ant-loving beetles, cave millipedes, and cave Gesneriaceae species have been reported (Figs 11–13; Gorochov et al. 2006; Lin and Li 2014; Yin et al. 2015; Tian et al. 2016, 2017, 2019; Song et al. 2017; Wang et al. 2017; Liu and Golovatch 2018; Deuve et al. 2020), but there are still many cave species remaining undiscovered, for example, the cave-dwelling rhaphidophorids covered in this study. Twenty-nine species of cave-dwelling rhaphidophorids have been reported in China. Guizhou province is the central area of karst distributions in southern China, where 18 cave-dwelling rhaphidophorids have been found (Gorochov et al. 2006; Rampini et al. 2008; Wen 2018; Feng et al. 2019, 2020; Qin et al. 2019; Zhou and Yang 2020; Zhu et al. 2020; Li et al. 2021; Zhu and Shi 2021). In this work, another ten new species are added to the Chinese fauna based on newly acquired material from Guizhou. This study further reveals the high degree of morphological similarity and cryptic diversity of species in the subgenus Gymnaeta, making it more challenging to delimitate these species using morphological characteristics. Furthermore, we agree with the views presented by Zhu et al. (2020) and confirm that Tachycines (Gymnaeta) aspes (Rampini & Di Russo, 2008) is a valid species and not a synonym of Tachycines (Gymnaeta) proximus (Gorochov, Rampini & Di Russo, 2006) according to the varying degrees of reduction of the fastigium vertices and eyes, the higher number of spines on the hind tibia, and the shape of male genitalia. Moreover, we consider that *Eutachycinescrenatus* (Gorochov, Rampini & Di Russo, 2006) should be transferred to the subgenus Gymnaeta, due to the following genitalic characteristics: median lobe that is shorter than lateral lobe and four lateral lobes that are not sclerotized.

## ﻿Materials and methods

All specimens in this article were collected in karst caves by hand, sometimes assisted with a swipe net, and preserved in 75% ethanol. Morphological characteristics were examined using an Olympus SZ61 stereomicroscope. The male genitalia was preserved in a solution of ethanol and glycerin. Photographs were taken by an Olympus DP22 digital camera and processed with Adobe Photoshop CS6. All specimens are deposited in the Institute of Karst Caves, Guizhou Normal University, Guizhou Province, China (**IKCGZNU**).

The morphological terms and classification follow Gorochov et al. (2006) and Qin et al. (2019).

## ﻿Taxonomy

### ﻿Genus *Tachycines*

#### Subgenus ﻿ Gymnaeta

Taxon classificationAnimaliaOrthopteraRhaphidophoridae

Adelung, 1902

34BD426E-8E78-574C-88DB-7ACD60789CAC

Gymnaeta Adelung, 1902. *Annuaire du Musée Zoologique de l’Académie Impériale des Sciences de St. Petersburg* 7: 62; Kirby 1906. A Synonymic Catalogue of Orthoptera 2: 125.
Diestrammena (Gymnaeta) : Jacobson, 1905[1902–1905]. In: Jacobson and Bianchi, *Orthopteroid and Pseudoneuropteroid Insects of Russian Empire and adjacent countries*, 329, 352, 434; Gorochov 1994. In: Gorochov and Kireichuk [Eds.], *Proceedings of the Zoological Institute of the Russian Academy of Sciences*, 257: 49; Otte 2000. Orthoptera Species File 8: 55; Jiao et al. 2008. *Zootaxa* 1917: 55; Zhang and Liu 2009. *Zootaxa* 2272: 21.
Tachycines (Gymnaeta) : Karny, 1934. Konowia 13 (1–3): 218; Karny 1937. *Genera Insectorum* 206: 248; Storozhenko 1990. *Entomologicheskoe Obozrenie* 69(4): 845, 847; Qin et al. 2018. *Zootaxa* 4374(4): 452; Qin et al. 2019. *Zootaxa* 4560(2): 274; Feng et al. 2019. *Zootaxa* 4674(4): 492; Zhou and Yang 2020. *ZooKeys* 937: 21–29; Zhu et al. 2020. *Zootaxa* 4809(1): 72.

##### Type species.

*Gymnaetaberezovskii* Adelung, by subsequent designation; authority: Kirby, W.F. 1906. A Synonymic Catalogue of Orthoptera (OrthopteraSaltatoria, Locustidae vel Acridiidae) 2: i–viii, 1–562.

#### ﻿ Tachycines (Gymnaeta) zhongi sp. nov.

Taxon classificationAnimaliaOrthopteraRhaphidophoridae

60754258-796F-5BB1-8733-3B64FFFA0213

http://zoobank.org/705C74CA-5D8A-46E1-8223-FDA318E27D72

Figs 1A–D , 15

##### Specimens examined.

***Holotype***, 1♂, Daxiao Dong, Xinchang township, Liuzhi Special District, 900 m, 2019-VII-28, collected by Jinhua Zhong, Xulin Zhou, Lingzhi Ou, Guang Wang, Benzhang Shi, Juan Liao and Liangfeng An; ***paratypes***, 5♂, 2♀, same collection data as for holotype.

##### Diagnosis.

**Figure 1.**
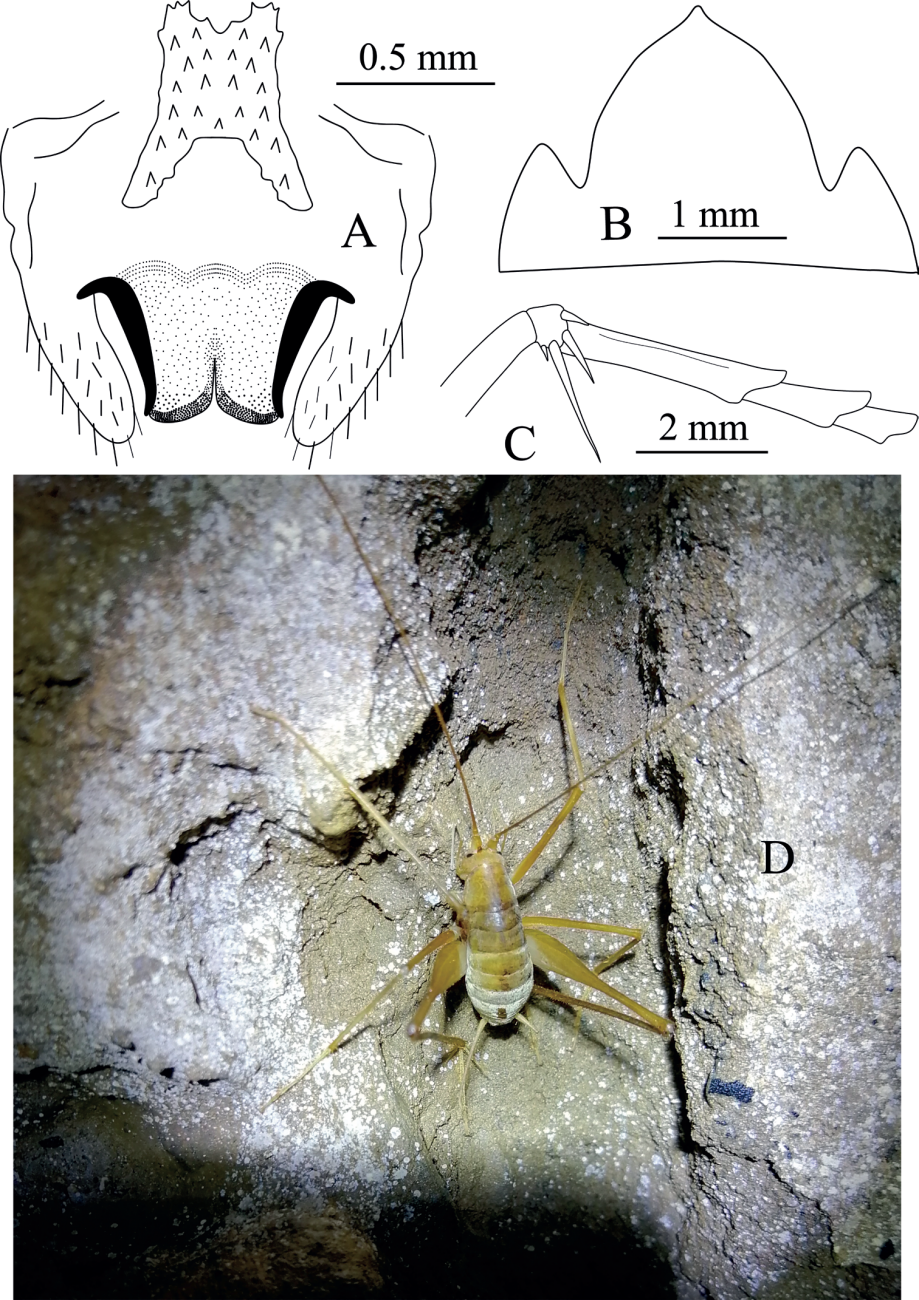
Tachycines (Gymnaeta) zhongi sp. nov., **A** male genitalia, dorsal view **B** female subgenital plate in ventral view **C** hind tarsus in lateral view **D** male live habitus dorsal view.

This new species is similar to T. (G.) caudatus (Gorochov et al., 2006) regarding the shape of the female subgenital plate, but the female subgenital plate of the new species has a small triangle on both sides, while the latter is without. Also similar to T. (G.) chenhui (Rampini & Di Russo, 2008) regarding the shape of the male epiphallus, but the new species is smaller, with its body length not exceeding 13 mm, vertex conical tubercles extremely reduced, scarce (Fig. 1D), ventral conical projections of 3^rd^–8^th^ abdominal sternites less developed, forming smaller and shorter projections, hind tarsus keeled ventrally; T. (G.) chenhui has a larger body exceeding 13 mm, vertex conical tubercles of intermediate development, ventral conical projections of 3^rd^–8^th^ abdominal sternites developed, forming larger and longer projections, hind tarsus with bristles ventrally.

##### Description.

**Male.** Body medium and small-sized (Fig. 1D). Eyes slightly reduced, ocelli absent; conical tubercles of vertex reduced. Legs elongate and slender; fore femur approx. 3.1–3.2 times longer than the pronotum, ventrally unarmed, the internal genicular lobe with single small spine, external genicular lobe with single elongate movable spur; ventral side of fore tibiae with one internal spur and two external spurs. Mid femur with an elongate movable spur on the inner and outer genicular lobes, ventrally unarmed; mid tibiae beneath with one internal spur and one external spur. Hind femur without ventral spine, internal genicular lobe with one small spine; hind tibiae dorsally on both sides with 23–25 spines, sparsely arranged. Supra-internal spur of hind tibiae not exceeding ventral apex of hind tarsus. Hind tarsus keeled ventrally and with one dorsal apical spine (Fig. 1C). Small and short ventral conical projections of 3^rd^–8^th^ abdominal sternites developed, but distal ones obtuse and densely ciliated. Cerci extremely long. Male genitalia with H-shaped epiphallus, middle lobe and lateral sclerites of genitalia almost at the same level at the bottom (Fig. 1A).

**Female.** Other characteristics are similar to the male. Subgenital plate with three lobes, median lobe large and nearly triangular (Fig. 1B). Ovipositor is slightly longer than half the length of hind femur.

##### Coloration.

Body uniformly yellowish brown.

##### Measurements

**(mm).** Body ♂11.2–11.6, ♀10.8–12.1; pronotum ♂3.5, ♀3.8; fore femur ♂11.1–11.5, ♀10.8–12.3; hind femur ♂18.5–19.3, ♀18.4–20.0, ovipositor 10.0–11.2.

##### Distribution of light zone.

Weak light and dark light zones.

##### Cave adaptation type.

Troglobite.

##### Etymology.

The specific epithet refers to the person’s last name who led us to collect the specimens.

#### ﻿ Tachycines (Gymnaeta) jinniui sp. nov.

Taxon classificationAnimaliaOrthopteraRhaphidophoridae

B188F142-7154-59F1-B65D-89387466F2E2

http://zoobank.org/144C1AF3-1041-4D1C-A340-C9C6832F74CC

Figs 2A , 14 , 15

##### Specimens examined.

***Holotype***, 1♀, Jinniu Cave (Fig. 14), Libo County, 2017-X-23, collected by Xulin Zhou, Dongshan Xu, Weicheng Yang; ***paratype***, 1♀, same collection data as for holotype.

##### Diagnosis.

The new species is very similar to Tachycines (Gymnaeta) trapezialis Zhou & Yang, 2020 but differs from the latter by having slightly reduced eyes and the conical tubercles of the vertex intermediately reduce, the hind tibia dorsally on each side has 78–85 spines instead of 54–60 spines.

**Figure 2.**
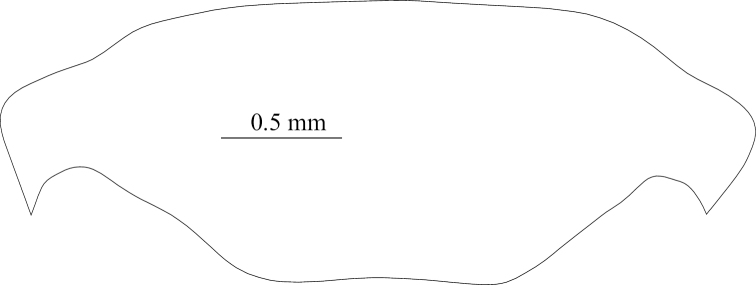
Tachycines (Gymnaeta) jinniui sp. nov., female, subgenital plate in ventral view.

##### Description.

**Female.** Body medium sized. Vertex conical tubercles slightly reduced, apex obtuse, ommateum black and well developed. Legs elongate and slender; fore femur approx. 2.5–2.7 times longer than the pronotum, ventrally unarmed, the internal genicular lobe with a small spine, external genicular lobe with one elongate movable spur; ventral side of fore tibiae with one internal spur and two external spurs. Mid femur with an elongate movable spur on internal and external genicular lobes, ventrally unarmed; mid tibiae beneath with two internal spurs and two external spurs. Hind femur without ventral spine, internal genicular lobe without spine; hind tibiae dorsally on both sides with 79–86 spines, arranged in groups. Supra-internal spur of hind tibiae not exceeding ventral apex of hind tarsus. Hind tarsus keeled ventrally, with one dorsal apical spine. Cerci long and slender. Ovipositor shorter than half length of hind femur.

**Male.** Unknown.

##### Coloration.

Body color uniform, yellowish brown, eyes black.

##### Measurements

**(mm).** Body ♀ 13.7–16.7; pronotum ♀ 5.1–5.3; fore femur ♀ 13.6–14.1; hind femur ♀ 25.2–26.0; ovipositor 8.4–8.5.

##### Distribution of light zone.

Dark light zone.

##### Cave adaptation type.

Troglophile.

##### Etymology.

The new species is named after the collection locality of the specimens (Jinniu cave).

#### ﻿ Tachycines (Gymnaeta) shibenzhangi sp. nov.

Taxon classificationAnimaliaOrthopteraRhaphidophoridae

3BB7D26E-9E70-59A3-BCA4-AA8803A11B57

http://zoobank.org/84644FCC-1440-4FF1-B00E-3D1A70F9450A

Figs 3A–D , 15

##### Specimens examined.

***Holotype***, 1♂, Xuehua Cave, Zhonghe Town, Sandu County, 2019-VII-28, collected by Xulin Zhou, Benchang Shi, Changzhen Zheng, Haixia Luo, Gui Liang, Hailian Lan, Panpan Ren and Juan Liao; ***paratypes***, 16♂, 15♀, same data as the holotype.

##### Diagnosis.

The characteristic of the male genitalia of the new species is distinct from that of other groups: the epiphallus of the male genitalia is semi-circular, and the lateral sclerites sub-elliptical. In addition, the conical tubercles of the vertex are absent, the ommateum are extremely degenerated, the mid tibiae ventrally without spur or spine, the ventral conical projections of 3^rd^–8^th^ abdominal sternites developed, and the distal ones are obtuse and densely ciliated.

##### Description.

**Figure 3.**
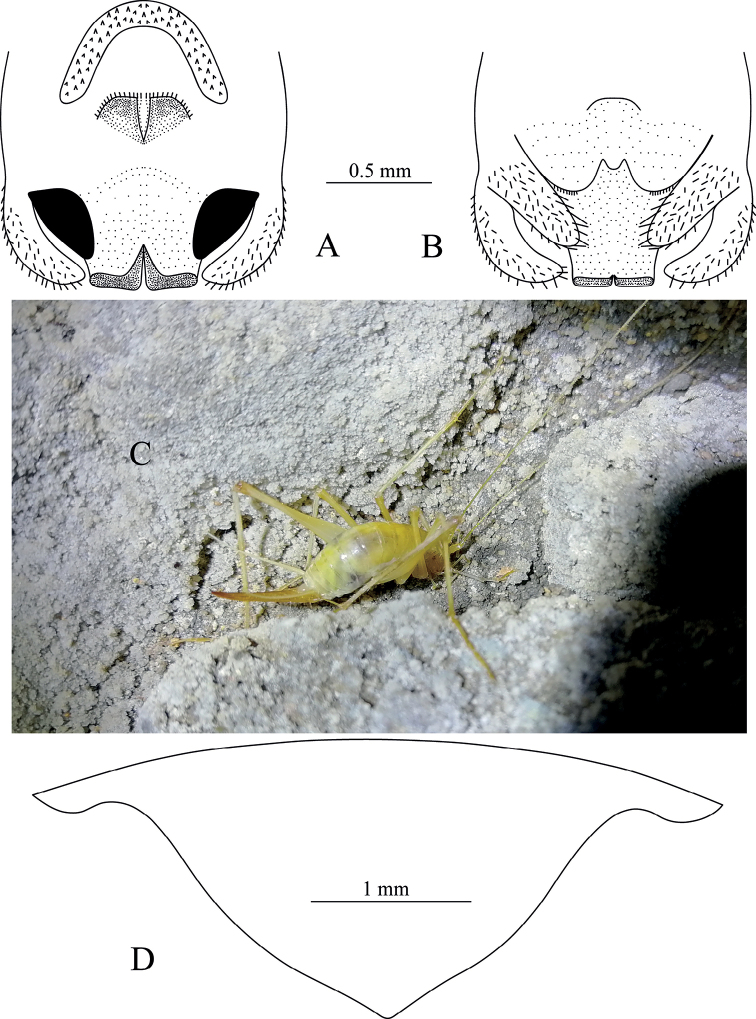
Tachycines (Gymnaeta) shibenzhangi sp. nov., **A** male genitalia in dorsal view **B** male genitalia in ventral view **C** female live habitus in dorsal view **D** female subgenital plate in ventral view.

**Male.** Body smaller than the average for the subgenus. Vertex conical tubercles absent, ommateum extremely degenerated, present by narrow stripes with several black facets (some individuals have no black facets and are completely blind). Legs elongate and slender, fore femur approx. 2.6–3.0 times longer than the pronotum, ventrally unarmed, external genicular lobe with one elongate movable spur, internal knee lobe without spine; fore tibiae beneath with one external spur (sometimes with two external spurs), but without internal spur. Mid femur with an elongate movable spur on both internal and external genicular lobes, ventrally unarmed; mid tibiae ventrally without internal or external spur. Hind femur without spines ventrally; hind tibiae dorsally with 11–18 inner spines and 13–18 outer spines, sparsely arranged. Supra-internal spur of hind tibiae not exceeding ventral apex of hind tarsus. Hind tarsus ventrally with bristles. Ventral conical projections of 3^rd^–8^th^ abdominal sternites developed, but distal ones obtuse and densely ciliated. Epiphallus of male genitalia nearly semi-circular, lateral sclerites sub-elliptical (Fig. 3A, B).

**Female.** Appearance is similar to the male. Subgenital plate nearly triangular, apical area slightly blunt. Ovipositor is longer than half length of hind femur, dorsal margin smooth, and apical area of ventral margin denticulate.

##### Coloration.

Body color uniform, pale yellow, abdomen slightly transparent and the internal organs are visible.

##### Measurements

**(mm).** Body ♂9.4–12.1, ♀11.2–12.5; pronotum ♂2.9–3.3, ♀ 3.0–3.2; fore femur ♂ 8.1–8.8, ♀ 8.2–9.6; hind femur ♂ 13.6–14.6, ♀ 13.6–14.8; ovipositor 9.1–10.0.

##### Distribution of light zone.

Dark light zone.

##### Cave adaptation type.

Troglobite.

##### Etymology.

The specific epithet refers to the name of the person who provided crucial help in collecting the specimens.

#### ﻿ Tachycines (Gymnaeta) lahaidensis sp. nov.

Taxon classificationAnimaliaOrthopteraRhaphidophoridae

A65AAC53-5476-5943-8742-B3D28404E9B5

http://zoobank.org/365EE275-1199-4EC4-A0BA-0CD676277EEE

Figs 4A–D , 15

##### Specimens examined.

***Holotype***, 1♂, Lahaide Dong, Pinglang Town, Duyun City, 2015-VII-24, collected by Qing Wen, Dongshan Xu, Yuanchan Yu, Yi Luo, Guang Zhang; ***paratypes***, 6♂, 8♀, same data as the holotype.

##### Diagnosis.

The new species is very similar to Tachycines (Gymnaeta) shibenzhangi sp. nov., as both species have an arc-shaped epiphallus. The difference is that the ventral surface of the hind tarsus keeled in the new species, but differs from the latter in that: lower notch of the epiphallus is rather small, hind tarsus keeled beneath, epiphallus of male nearly n-shaped; ventral conical projections of 3^rd^–8^th^ abdominal sternites developed, apex mucronate without dense cilia.

##### Description.

**Male.** Body medium sized. Vertex conical tubercles inconspicuous, eyes moderately reduced, approx. 1/2 the size of the normal eye. Legs elongate, slender; fore femur approx. 2.1–2.5 times longer than the pronotum, ventrally unarmed, external genicular lobe with one elongated movable spur, internal knee lobe without spine; fore tibiae ventrally with two external spurs and one internal spur. Mid femur with an elongate movable spur on the internal and external genicular lobes, ventrally unarmed; mid tibiae beneath with one external spur and one internal spur. Hind femur without spine ventrally; hind tibiae dorsally with 27–30 internal spines and 22–26 external spines, sparsely arranged. Supra-internal spur of hind tibiae not exceeding the ventral apex of hind tarsus. Hind tarsus keeled ventrally. Ventral conical projections of 3^rd^–8^th^ abdominal sternites developed, distally mucronate without cilia. Epiphallus of male genitalia nearly n-shaped, lateral sclerites distinctly long and narrow.

**Figure 4.**
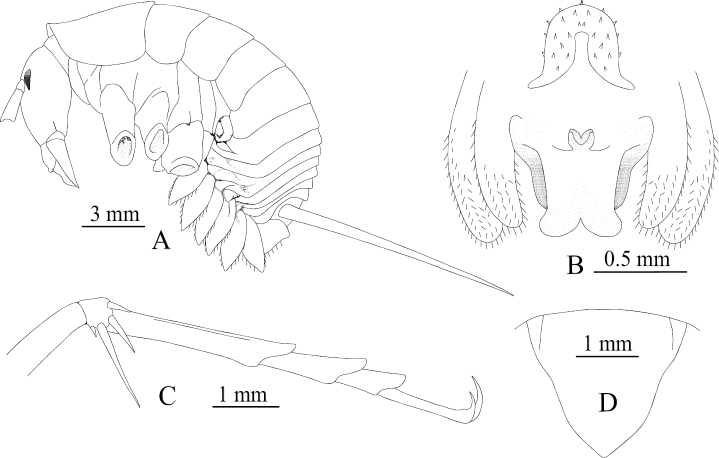
Tachycines (Gymnaeta) lahaidensis sp. nov., **A** male body in lateral view **B** male genitalia in dorsal view **C** hind tarsus in lateral view **D** female subgenital plate in ventral view.

**Female.** Appearance is similar to the male. The subgenital plate is nearly triangular, its apical area slight obtuse (Fig. 4D). Ovipositor is longer than half length of the hind femur, dorsal margin smooth, apical area of ventral margin denticulate, bent slightly upwards.

##### Coloration.

Body brown, ovipositor wheat.

##### Measurements

**(mm).** Body ♂ 10.5–12.3, ♀ 11.2–12.6; pronotum ♂ 4.0–4.8, ♀ 3.8–3.9; fore femur ♂ 10.2–10.30, ♀ 10.1–10.3; hind femur ♂ 18.9–19.0, ♀ 18.0–18.1; ovipositor 10.0–11.0.

##### Distribution of light zone.

Dark light zone.

##### Cave adaptation type.

Troglobite.

##### Etymology.

The specific epithet refers to the Lahaide cave.

#### ﻿ Tachycines (Gymnaeta) pinglangus sp. nov.

Taxon classificationAnimaliaOrthopteraRhaphidophoridae

C7DDA052-CD52-5193-B7EA-FD3DC8AB7658

http://zoobank.org/C2D9C063-9A83-464F-8EE2-EDDD561C4745

Figs 5A–D , 15

##### Specimens examined.

***Holotype*** 1♂, Lagaobieran Dong, Pinglang Town, Duyun City, 2015-VII-25, collected by Qing Wen; ***paratypes***, 11♂, 16♀, 2015-VII-25, collected by Qing Wen, Dongshan Xu, Yi Luo, Yuanchan Yu, Guang Zhang.

##### Diagnosis.

The new species is very similar to Tachycines (Gymnaeta) ferecaecus (Gorochov, Rampini & Di Russo, 2006): both species have a nearly quadrate-shaped epiphallus, but the new species can be distinguished from the latter by the absence of an ommateum (without any black facets), only the base of the ommateum was faintly visible.

##### Description.

**Figure 5.**
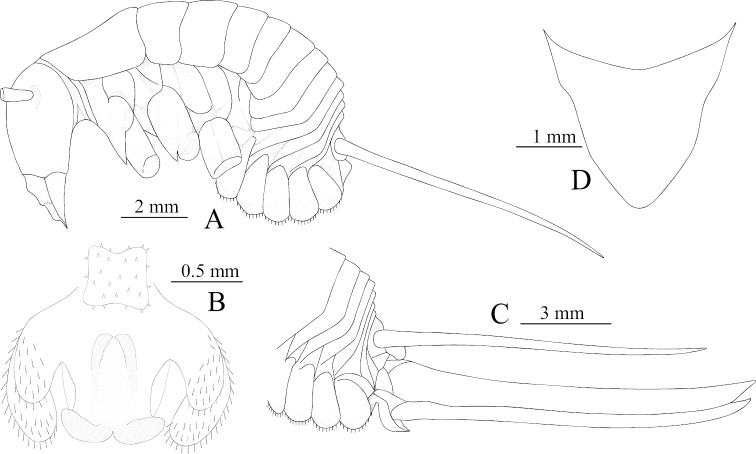
Tachycines (Gymnaeta) pinglangus sp. nov., **A** male body in lateral view **B** male genitalia in dorsal view **C** female terminal in lateral view **D** female subgenital plate in ventral view.

**Male.** Body medium sized in the subgenus (Fig. 5A). Vertex conical tubercles almost absent, ommateum completely reduced (appears to be without any black facets, only ommateum base); Legs elongate and slender; fore femur approx. 2.8–3.2 times longer than the pronotum, ventrally unarmed, external genicular lobe with one elongate movable spur, internal knee lobe without spine; fore tibiae beneath with one external spur (sometimes with two external spurs), but without internal spur. Mid femur with an elongate movable spur on the internal and external genicular lobes, ventrally unarmed; mid tibiae ventrally with one external spur and one internal spur. Hind femur without spines ventrally; hind tibiae dorsally with 12–15 internal spines and 12 or 13 external spines, sparsely arranged. Supra-internal spur of hind tibiae shorter than the ventral apex of hind tarsus. Hind tarsus with bristles ventrally. Ventral conical projections of 3^rd^–8^th^ abdominal sternites developed, distally obtuse, and densely ciliated. Epiphallus of male genitalia nearly quadrate, median lobe of genitalia with a pair of wide apical lobules, but without distinct lateral sclerites (Fig. 5B).

**Female.** Appearance is similar to the male. The subgenital plate is nearly triangular, and the apical area slightly obtuse. Ovipositor is longer than half of the hind femur length, brown, dorsal margin smooth, apical area of ventral margin denticulate, bent slightly upwards.

##### Coloration.

Body yellowish brown, ovipositor wheat.

##### Measurements

**(mm).** Body ♂ 9.5–13.0, ♀ 8.2–12.0; pronotum ♂ 3.2–3.8,♀ 3.2–3.4; fore femur ♂ 10.2–10.50, ♀ 10.2–10.4; hind femur ♂ 13.2–14.7, ♀ 13.2–14.6; ovipositor 8.0–10.4.

##### Distribution of light zone.

Dark light zone.

##### Cave adaptation type.

Troglobite.

##### Etymology.

The specific epithet refers to the locality where the type specimens were collected.

#### ﻿ Tachycines (Gymnaeta) shanduensis sp. nov.

Taxon classificationAnimaliaOrthopteraRhaphidophoridae

9440300A-AA5B-5DFC-A7A4-AC9E901732D2

http://zoobank.org/ED670C87-E4D5-48C1-A93C-83949F4F8FC3

Figs 6A–D , 15

##### Specimens examined.

***Holotype*** 1♂, Shuilong Cave, Sandu County, 2019-VII-22, collected by Xulin Zhou, Benchang Shi, Changzhen Zheng, Gui Liang, Haixia Luo, Hailian Lan, Juan Liao; ***paratypes***, 6♂, 8♀, same data as holotype.

##### Diagnosis.

This species is rather similar to Tachycines (Gymnaeta) solida (Gorochov, Rampini & Di Russso, 2006) and Tachycines (Gymnaeta) tongrenus Feng, Huang & Luo, 2020, but the male epiphallus of the new species has a distal shallow notch clearly wider than the upper notch, the median process of the male genitalia is significantly longer than the lateral sclerites, hind tibiae dorsally on both sides with 34–46 spines, hind tarsus keeled beneath; however, in Tachycines (Gymnaeta) solida, the male epiphallus has the upper and lower notches almost the same size, hind tibiae dorsally on both sides with 62–69 spines; in Tachycines (Gymnaeta) tongrenus, the hind tibia dorsally with 48–49 inner spines and 54–56 outer spines, hind tarsus with bristles ventrally.

##### Description.

**Figure 6.**
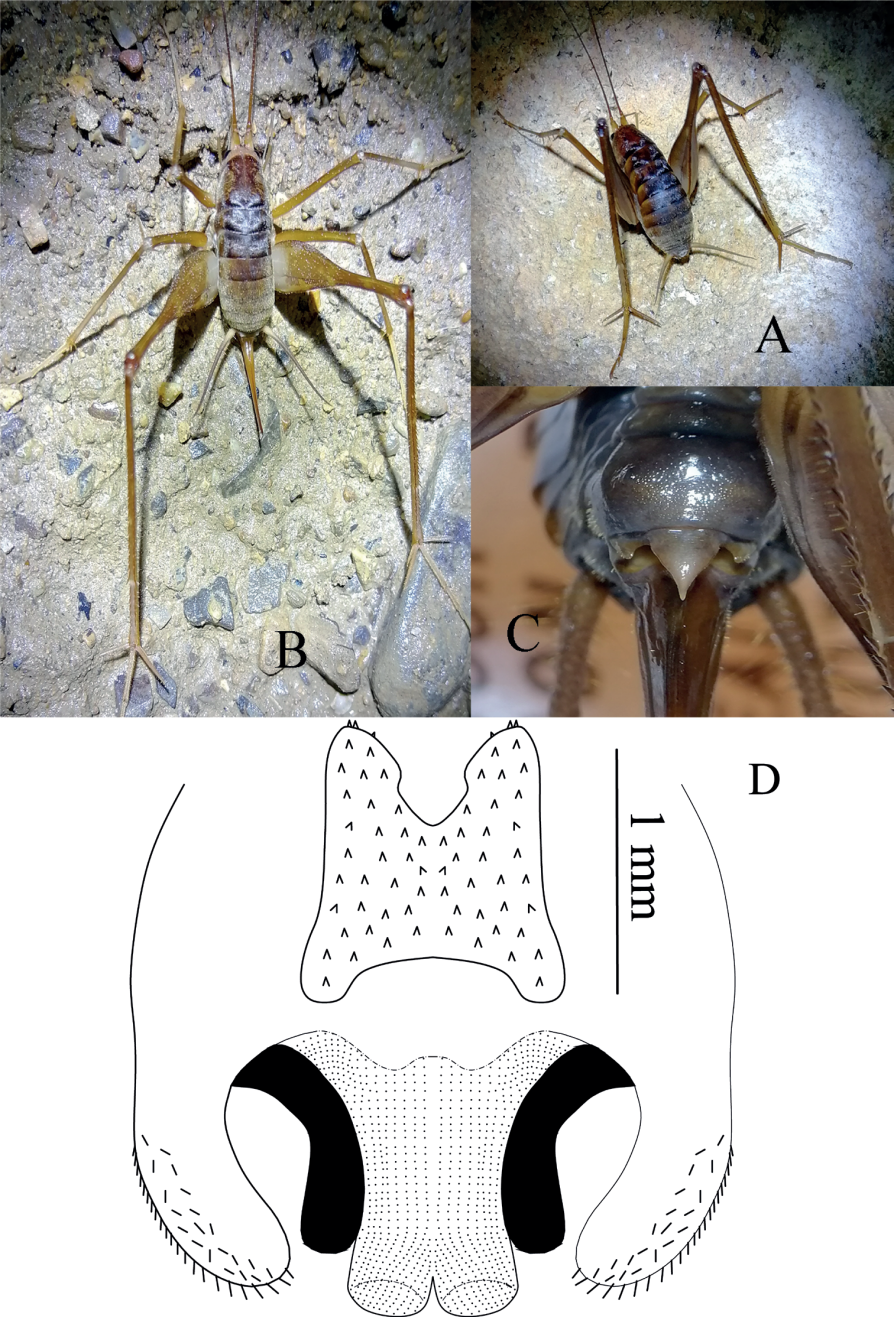
Tachycines (Gymnaeta) shanduensis sp. nov., **A** male live habitus in dorsal view **B** female live habitus in dorsal view **C** female subgenital plate in ventral view **D** male genitalia in dorsal view.

**Male.** Body rather large for this subgenus. Vertex conical tubercles are well developed, bisected from the base; ommateum is black and well developed (Fig. 6A). Legs elongate and slender; fore femur approx. 1.9–2.1 times longer than the pronotum, ventrally unarmed, external genicular lobe with one elongate movable spur, internal knee lobe with a small spine; fore tibiae beneath with two external spurs and one internal spur. Mid femur with an elongate movable spur on both internal and external genicular lobes, ventrally unarmed; mid tibiae beneath with one external spur and one internal spur. Hind femur without spines ventrally; hind tibiae dorsally with 34–43 internal spines and 38–46 external spines, arranged in groups. Supra-internal spur of hind tibiae shorter than the dorsal apex of hind tarsus. Hind tarsus keeled ventrally, with one dorsal apical spine. Epiphallus of male genitalia nearly H-shaped, lateral sclerites distinctly long and narrow, upper notch rather smaller than lower notch.

**Female.** Appearance is similar to male (Fig. 6B). Subgenital plate with three lobes, median lobe large, triangular, and apical area sharp (Fig. 6C). Ovipositor is shorter than half of the hind femur length, dorsal margin smooth, apical area of ventral margin denticulate.

##### Coloration.

Body dark brown, mixed with tawny stripes, hind femur with brown diagonal stripe.

##### Measurements

**(mm).** Body ♂ 17.3–19.5, ♀17.3.0–19.6; pronotum ♂ 6.8–7.6, ♀ 66–7.3; fore femur ♂ 13.3–15.1, ♀ 13.8–14.6; hind femur ♂ 28.5–31.9, ♀ 28.4–30.7; ovipositor 12.8–13.5.

##### Distribution of light zone.

Light zone, weak light zone, and dark light zone.

##### Cave adaptation type.

Troglophile.

##### Etymology.

The name of the new species refers to the type locality.

#### ﻿ Tachycines (Gymnaeta) buyii sp. nov.

Taxon classificationAnimaliaOrthopteraRhaphidophoridae

BDAF810C-D431-5DBC-A705-A47FDC7F974D

http://zoobank.org/935DF22A-FD7C-4C2C-80FF-04743039FDE2

Figs 7A–C , 15

##### Specimens examined.

***Holotype*** 1♂, Sanjiaoshan Cave, Ziyun County, 2019-X-2, collected by Xulin Zhou, Haixia Luo, Panpan Ren, Meizhen Deng and Suqin Zhao; ***paratypes***, ♂15, ♀18, same data as holotype.

##### Diagnosis.

This new species is rather similar to Tachycines (Gymnaeta) solida (Gorochov, Rampini & Di Russo, 2006), but differs as follows: the new species epiphallus of male genitalia with upper notch smaller and shallower than lower notch, hind tarsus ventrally with bristles; in T. (G.) solida the epiphallus of the male genitalia with upper notch and lower notch almost the same size, hind tarsus keeled ventrally.

**Figure 7.**
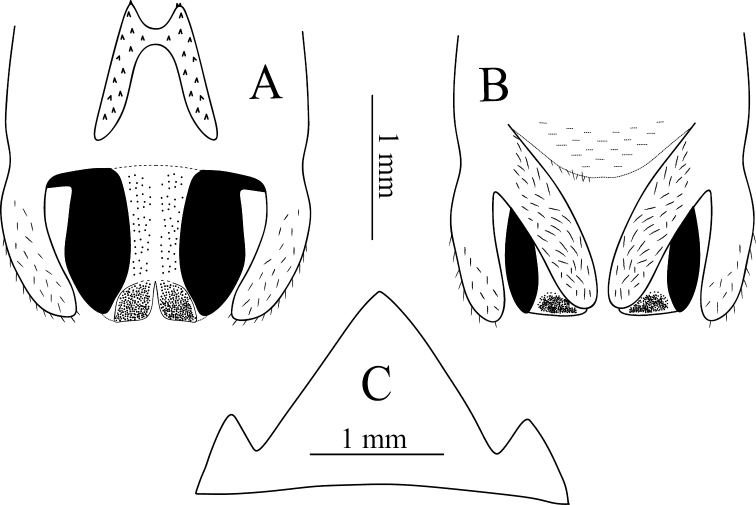
Tachycines (Gymnaeta) buyii sp. nov., **A** male genitalia in dorsal view **B** male genitalia in ventral view **C** female subgenital plate in ventral view.

**Description**. **Male.** Body rather small for this subgenus. Vertex conical tubercles well-developed, bisected from the base; ommateum black and well developed. Legs elongate and slender; fore femur 2.0–2.1 times longer than the pronotum, ventrally unarmed, external genicular lobe with one elongate movable spur, internal knee lobe without spine; fore tibiae beneath with two external spurs and one internal spur. Mid femur with an elongate movable spur on both internal and external genicular lobes, ventrally unarmed; mid tibiae beneath with one external spur and one internal spur. Hind femur without spines ventrally; hind tibiae dorsally with 35–44 internal spines and 36–46 external spines, arranged in groups. Supra-internal spur of hind tibiae not exceeding the ventral apex of hind tarsus. Hind tarsus ventrally with bristles. Epiphallus of male genitalia nearly H-shaped, lower notch rather deeper than upper notch (Fig. 7A, B).

**Female.** Appearance is similar to the male. Subgenital plate with three lobes, median lobe large and triangular. Ovipositor is slightly shorter than half of the hind femur length, dorsal margin smooth, apical area of ventral margin denticulate.

##### Coloration.

Body brown, mixed with dark brown patches. Hind femur with brown stripe, and dark brown rings located at 2/3 of the length.

##### Measurements

**(mm).** Body ♂10.5–11.3, ♀10.8–11.5; pronotum ♂3.9–4.5, ♀4.1–4.6; fore femur ♂ 8.3–8.9, ♀ 8.4–9.3; hind femur ♂ 14.7–16.5, ♀ 15.3–17.8; ovipositor 7.2–8.6.

##### Distribution of light zone.

Light zone, weak light zone, and dark light zone.

##### Cave adaptation type.

Troglophiles.

##### Etymology.

The specific epithet refers to the native BuYi people who have lived in southern Guizhou for generations.

#### ﻿ Tachycines (Gymnaeta) portae sp. nov.

Taxon classificationAnimaliaOrthopteraRhaphidophoridae

650CBEF2-1CDE-5D7C-87FA-819DA248AB34

http://zoobank.org/6A1D6FF0-A4CC-4921-9E0B-0E6EDFC09E7E

Figs 8A–C , 15

##### Specimens examined.

***Holotype*** 1♂, Niujingchongzi Dong Weining County, 2019-VII-17, collected by Xulin Zhou, Lingzhi Ou, Guang Wang, Rongxiang Su Benzhang Shi, Juan Liao and Liangfeng An. ***paratypes***, 4♂, 2♀, same data as holotype.

##### Diagnosis.

The new species is most closely related to Tachycines (Gymnaeta) buyii sp. nov., but it can be distinguished from the latter by the structure of epiphallus, and hind tarsus keeled ventrally.

##### Description.

**Figure 8.**
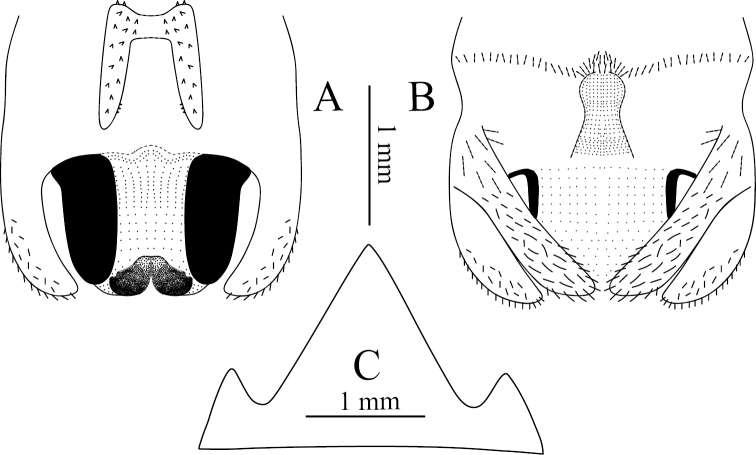
Tachycines (Gymnaeta) portae sp. nov., **A** male genitalia in dorsal view **B** male genitalia in ventral view **C** female subgenital plate in ventral view.

**Male.** Body rather small for this subgenus. Vertex conical tubercles well developed, bisected from the base; ommateum black and well developed. Legs elongate and slender; fore femur 1.8–1.9 times longer than the pronotum, ventrally unarmed, external genicular lobe with one elongate movable spur, internal knee lobe with a small spine; fore tibiae beneath with two external spurs and one internal spur. Mid femur with an elongate movable spur on both internal and external genicular lobes, ventrally unarmed; mid tibiae beneath with one external spur and one internal spur. Hind femur without spines ventrally; hind tibiae dorsally with 65–81 internal spines and 63–81 external spines, arranged in groups. Supra-internal spur of hind tibiae not exceeding the ventral apex of hind tarsus. Hind tarsus keeled ventrally. Epiphallus of male genitalia nearly door-shaped, lower notch rather deeper than upper notch (Fig. 8A, B).

**Female.** Appearance is similar to the male. Subgenital plate with three lobes, median lobe large triangular (Fig. 8C); ovipositor is slightly longer than half of the hind femur length, dorsal margin smooth, apical area of ventral margin denticulate.

##### Coloration.

Body brown, mixed with brown patches; hind femur with brown stripe.

##### Measurements

**(mm).** Body ♂6.0–7.0, ♀6.3–7.0; pronotum ♂4.0–4.5, ♀3.8–4.5; fore femur ♂6.8–8.0, ♀6.5–7.5; hind femur ♂10.0–11.5, ♀10.5–11.5; ovipositor 5.5–6.0.

##### Distribution of light zone.

Light and weak light zone.

##### Cave adaptation type.

Troglophile.

##### Etymology.

The specific epithet refers to the shape of epiphallus, the Latin word *porta* meaning door.

#### ﻿ Tachycines (Gymnaeta) ziyunensis sp. nov.

Taxon classificationAnimaliaOrthopteraRhaphidophoridae

7243602A-9FA7-5E5E-AB7A-0259E41F1E5D

http://zoobank.org/53F85BED-D0B8-46F5-AC23-0DE66CF2C3F1

Figs 9A–E , 15

##### Specimens examined.

***Holotype*** 1♂, Sanjiaoshan cave, Ziyun County, 2019-X-2, collected by Xulin Zhou, Haixia Luo, Panpan Ren, Meizhen Deng and Suqin Zhao, ***paratypes*** 15♂, 38♀, same data as holotype.

##### Diagnosis.

The new species is rather similar to Tachycines (Gymnaeta) shibenzhangi sp. nov., it can easily be distinguished by the eyes moderately reduced, ventral conical projections of 3^rd^–8^th^ abdominal sternites developed, distal mucronate without ciliated; but the latter of eyes extremely reduce, ventral conical projections of 3^rd^–8^th^ abdominal sternites developed, distal obtuse and densely ciliated.

##### Description.

**Figure 9.**
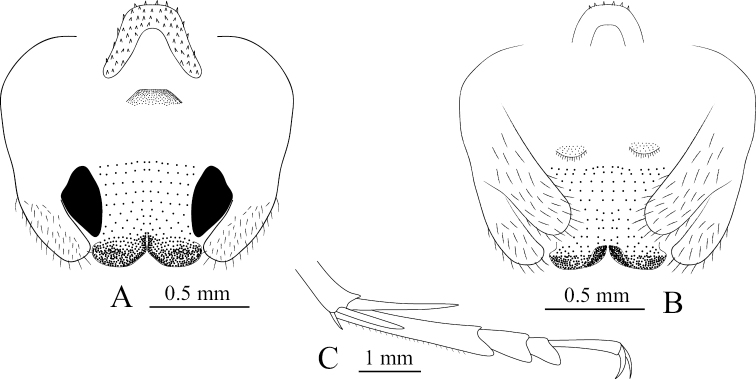
Tachycines (Gymnaeta) ziyunensis sp. nov., **A** male genitalia in dorsal view **B** male genitalia in ventral view **C** hind tarsus in lateral view **D** male live habitus in dorsal view **E** nymph of male, living habitus in dorsal view.

**Male.** Body medium size (Fig. 9D, E). Vertex conical tubercles almost absent, ommateum moderately reduced. Legs elongate and slender; fore femur approx. 2.5–3.1 times longer than the pronotum, ventrally unarmed, external genicular lobe with one elongate movable spur, internal knee lobe with a small spine; fore tibiae beneath with one external spur and one internal spur. Mid femur with an elongate movable spur on both internal and external genicular lobe, ventrally unarmed; mid tibiae beneath without internal and external spur. Hind femur without spines ventrally; hind tibiae dorsally with 9–15 internal spines and 9–13 external spines, sparsely arranged, supra-internal spur of hind tibiae not exceeding the ventral apex of hind tarsus. Hind tarsus ventrally with bristles (Fig. 9C). ventral conical projections of 3^rd^–8^th^ abdominal sternites developed, distal mucronate without cilia. Epiphallus of male genitalia nearly semi-circular, lateral sclerites sub-elliptical; median process of male genitalia with semi-sclerotized lobules at apical part and divided into two lobes, significantly longer than lateral sclerites (Fig. 9A, B).

**Female.** Appearance is similar to the male. Subgenital plate with three lobes, median lobe large, triangular; ovipositor is slightly longer than half of the hind femur length.

##### Coloration.

The body color is yellowish, face without dark brown stripes, uniformly pale yellow, ventral conical projections of abdominal sternites shiny white. Ovipositor is brownish yellow.

##### Measurements

**(mm).** Body ♂11.4–12.8, ♀12.2–13.2, pronotum ♂ 3.5–4.3, ♀ 3.9–4.3, fore femur ♂ 11.0–12.1, ♀ 10.2–10.8, hind femur ♂ 17.9–19.2, ♀ 16.9–17.9; ovipositor 8.9–10.3.

##### Distribution of light zone.

Dark light zone.

##### Cave adaptation type.

Troglobite.

##### Etymology.

The name of the new species refers to the type locality.

#### ﻿ Tachycines (Gymnaeta) jialiangensis sp. nov.

Taxon classificationAnimaliaOrthopteraRhaphidophoridae

BCC3032F-587C-5ACF-AC68-103EB72D4DFE

http://zoobank.org/68C1DDDD-C8AD-432B-9BBD-85D4610A79A5

Figs 10A–C , 15

##### Specimens examined.

***Holotype*** 1♂, Lajilou Cave, Jialiang Town, Libo County, 2017-X-23, collected by Xulin Zhou, Dongshan Xu, Weicheng Yang; ***paratypes***, 4♂, 2♀, same data as holotype.

##### Diagnosis.

This species is similar to T. (G.) ziyunensis sp. nov. but differs in that the hind tarsus ventrally bears bristles in T. (G.) ziyunensis sp. nov., and by the hind tibiae armed with 9–15 spines on both sides; however, the hind tarsus is keeled ventrally in T. (G.) jialiangensis sp. nov., and the hind tibiae are provided with 17–25 spines on both sides.

##### Description.

**Figure 10.**
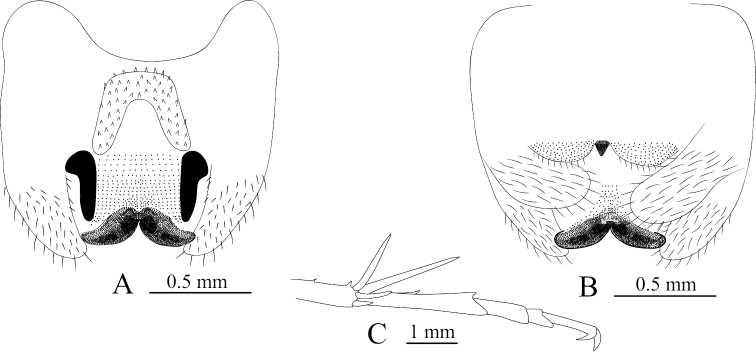
Tachycines (Gymnaeta) jialiangensis sp. nov., **A** male genitalia in dorsal view **B** male genitalia in ventral view **C** hind tarsus in lateral view.

**Male.** Body medium in size. Vertex conical tubercles almost absent, ommateum moderately reduced. Legs elongate and slender; fore femur 2.9–3.0 times longer than the pronotum, ventrally unarmed, external genicular lobe with single elongate movable spur, internal knee lobe without spine; fore tibiae beneath with two external spurs and one internal spur. Mid femur with an elongate movable spur on both internal and external genicular lobes, ventrally unarmed; mid tibiae beneath with an external spur and without internal spur. Hind femur without spines ventrally; hind tibiae dorsally with 18–25 internal spines and 17 or 18 external spines, sparsely arranged. Supra-internal spur of hind tibiae not exceeding ventral apex of hind tarsus. Hind tarsus keeled ventrally (Fig. 10C). ventral conical projections of 3^rd^–8^th^ abdominal sternites developed, distally mucronate without cilia. Epiphallus of male genitalia nearly semi-circular, lateral sclerites sub-elliptical, median process of male genitalia with semi-sclerotized lobules at apical part and divided into two lobes, significantly longer than lateral sclerites (Fig. 10A, B).

**Female.** Appearance is similar to the male. Subgenital plate with three lobes, median lobe large, triangular; ovipositor slightly shorter than half of the hind femur length, dorsal margin smooth, apical area of ventral margin denticulate.

##### Coloration.

Body color uniform, pale yellow; face without dark brown stripes; ventral conical projections of 3^rd^–8^th^ abdominal sternites shiny white.

##### Measurements

**(mm).** Body ♂ 11.8–12.0, ♀ 11.2–13.0; pronotum ♂ 3.5–3.6, ♀ 3.4–4.2; fore femur ♂ 10.3–10.6, ♀10.4–13.2; hind femur ♂17.5–18.4, ♀17.1–21.66, ovipositor 8.3–9.9.

##### Distribution of light zone.

Dark light zone.

##### Cave adaptation type.

Troglobite.

##### Etymology.

The name of the new species refers to the type locality.

## ﻿Discussion

Environmental conditions typical of the habitat deep within caves include the complete absence of light, a stable and usually very high humidity (> 95%), relatively low levels of available nutrients, and a nearly constant temperature (Taylor 2004). Cave organisms have been evolving in unusual and fascinating habitats, and the nature of these seems to be the loss of some structures (generally of eyes and pigment), such as in cavefish, cave-dwelling rhaphidophorids, and cave-adapted ground beetles. These organisms successfully navigate within such environments, find and capture food, identify and reproduce with conspecifics, and compete with one another for resources, all in the absence of visual cues. During the adaptive evolution in a cave environment, these cave-dwellers have evolved morphological, physiological, and behavioral modifications, many of which could promote their success lives in constant darkness (William et al. 2019).

**Figure 11.**
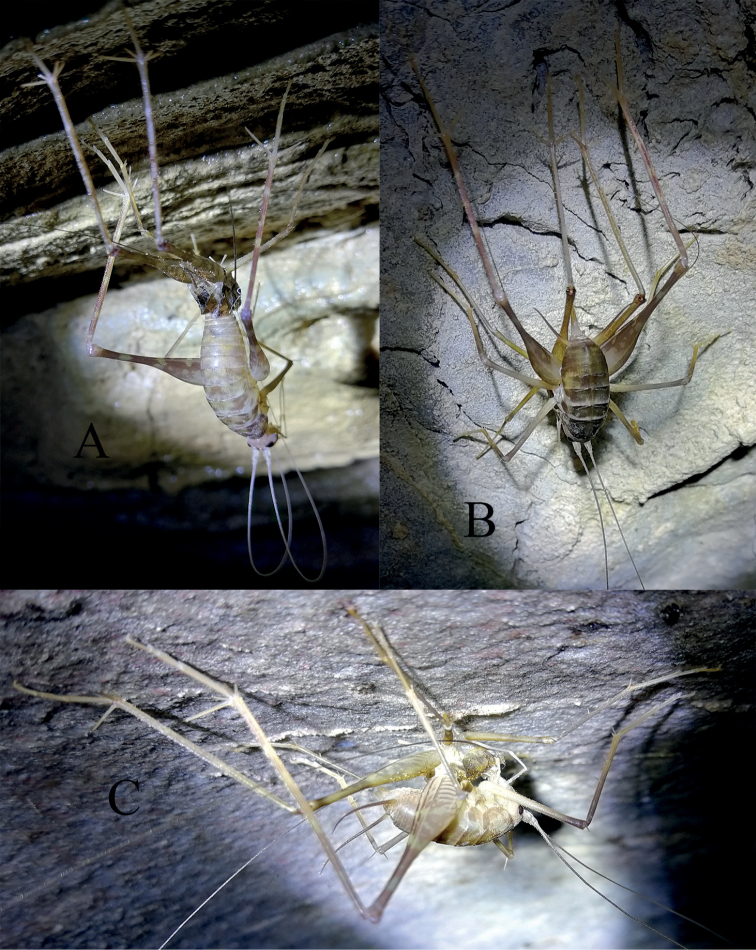
The ecdysial Tachycines (Gymnaeta) solida (Gorochov, Rampini & Di Russo, 2006). **A** in cave Donggou Dong **B, C** in cave Yu Dong.

There are two subfamilies of Rhaphidophoridae, Aemodogryllinae and Rhaphidophorinae, occurring in East Asia. Among them, species of the subgenus Gymnaeta are widely adapted to cave ecosystems, and belong to the Aemodogryllinae. The distribution of cave species of *Gymnaeta* is consistent with that of karst landforms, while the surface species are widely distributed in East Asia, ranging from forests to swamps, deserts, and the Tibet Plateau. According to our observation of many years, the surface-dwelling species of *Gymnaeta* usually demonstrate the following characteristics: a larger body size, darker body coloration, the ommateum and conical tubercles on the vertex well-developed, abdominal sternites without ventral projections, ventral spurs of the fore tibiae and mid tibiae well developed, the dorsal spines of hind tibiae dark and arranged in dense clusters, and the hind femur muscles well-developed, and hence these species are physically agile and good jumpers. Surface-dwelling species of *Gymnaeta* are widely distributed throughout China.

**Figure 12.**
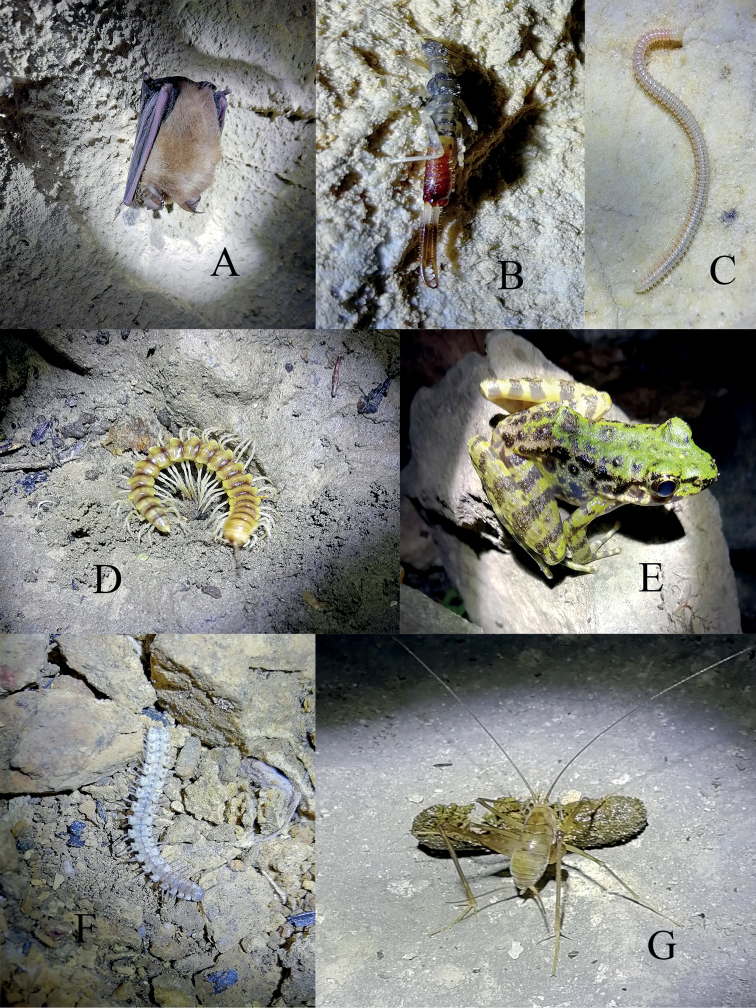
Other species found in the caves **A***Rhinolophus* sp. (Chiroptera, Rhinolophidae) in cave Donggou Dong **B** a running earwig in cave Xuehua Dong **C***Glyphiulus* sp. in cave Xuehua Dong **D** a Polydesmida in cave Da Dong **E** an *Odorrana* sp. in cave Wuming Dong near Banzhu town **F** an *Epanerchodus* millipede in cave Sanjiaoshan Dong **G** a Tachycines (Gymnaeta) sp. nymph feeding on rat droppings from Donggou Dong.

In contrast, the cave-adapted species of the subgenus have the ommateum and conical tubercles of the vertex reduced, the body appears thinner and smaller, the legs appear more slender, the ventral spurs of fore tibiae and mid tibiae are reduced and sometimes absent, the dorsal spines of hind tibiae are not pigmented and are sparse, the hind femur muscles are not obvious, and the ventral projections of abdominal sternites are developed. The cave-dwelling *Gymnaeta* are not good at jumping, as they have less developed legs muscles, leading them to almost walking on the ground like cave beetles when in danger. They are mainly distributed in karst areas of south China. We think that these morphological changes have occurred through adaptive evolution to cave environments. In addition, we consider the presence or absence of cilia on the distal of abdominal sternites projections in cave-adapted species of the subgenus Gymnaeta an important taxonomic characteristic. While these species in Chinese karst caves may be morphologically similar to surface-dwelling species, we suggest they may be genetically distinct, potentially representing different subspecies or lineages and we expect our future work to expand on these theories.

**Figure 13.**
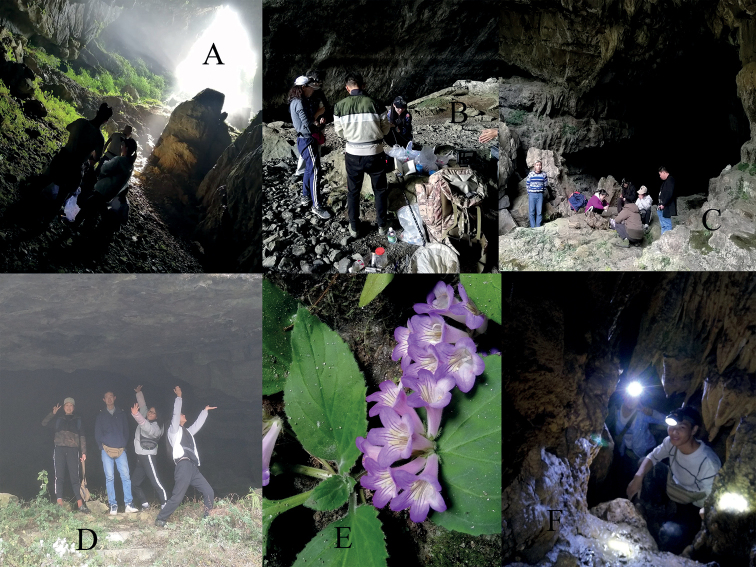
**A** Entrance of Ban Dong **B** entrance of Mawan Dong from Fuyan town **C** Entrance of Mawan Dong from Lengjiagou **D** entrance of Donggou Dong **E***Primulinaeburnea* in entrance of Ban Dong **F** two collectors in a small humid passageway in cave Sanjiaoshan Dong, showing the habitat where Tachycines (Gymnaeta) ziyunensis sp. nov. was collected.

## ﻿Conservation suggestions for China’s karst cave habitats and species

Caves and their associated ecosystems (mostly karst) represent resources of great value. These values can be grouped into three general clusters: ecological-scientific, economic, and cultural. Cave habitats and species are attracting increasing interest and concern among conservationists, cavers, and speleobiologists, and for good reason. Most troglobionts are highly restricted geographically and often are numerically rare, making them vulnerable to even relatively minor disturbances. As cave organisms are an essential part of biodiversity, it is hoped that we can raise public awareness and take adequate measures to protect karst cave ecosystems.

**Figure 14.**
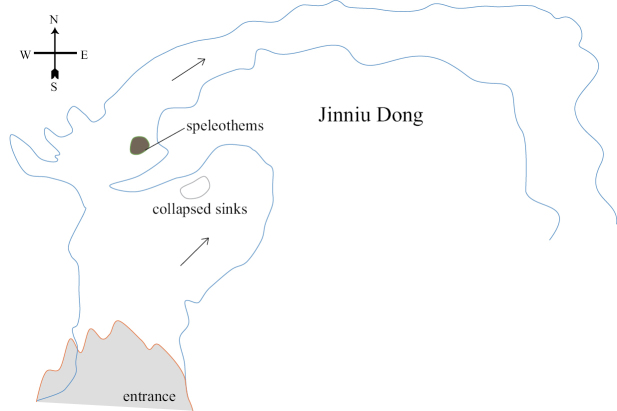
Map of Cave Jinniu Dong, type locality of Tachycines (Gymnaeta) jinniui sp. nov. The arrowhead indicates where in the cave system the rhaphidophorids were found.

In China, we have a rich diversity of troglobionts, but the biodiversity of Karst caves in China is under serious threat. We should choose high value and high priority caves, which need emergency attention in relation to protection, management, and conservation actions in the karst region of China. Such a fact does not exclude the need for the conservation of the other caves that should require attention, management, and/or conservation plans coordinated by the environmental supervisory agencies.

**Figure 15.**
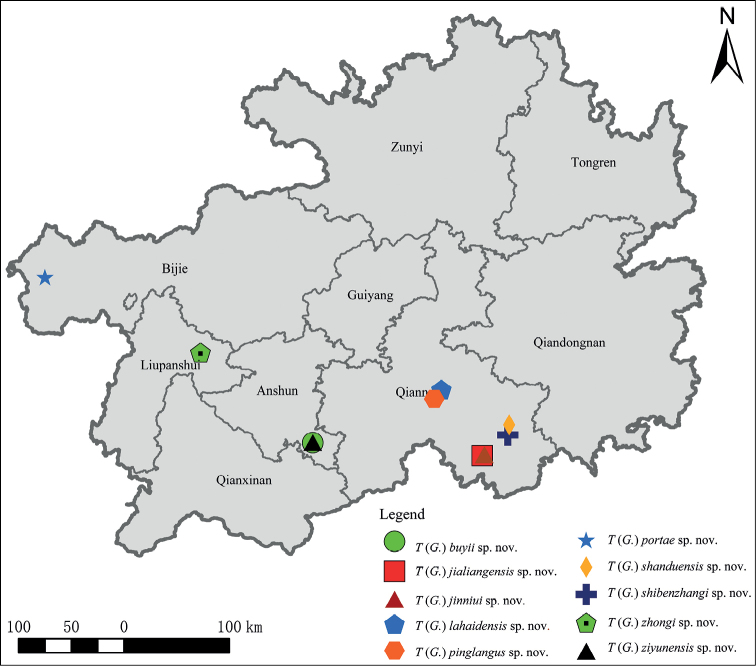
Distribution map for new species of subgenus Gymnaeta from Guizhou mentioned in this study.

## Supplementary Material

XML Treatment for Gymnaeta

XML Treatment for Tachycines (Gymnaeta) zhongi

XML Treatment for Tachycines (Gymnaeta) jinniui

XML Treatment for Tachycines (Gymnaeta) shibenzhangi

XML Treatment for Tachycines (Gymnaeta) lahaidensis

XML Treatment for Tachycines (Gymnaeta) pinglangus

XML Treatment for Tachycines (Gymnaeta) shanduensis

XML Treatment for Tachycines (Gymnaeta) buyii

XML Treatment for Tachycines (Gymnaeta) portae

XML Treatment for Tachycines (Gymnaeta) ziyunensis

XML Treatment for Tachycines (Gymnaeta) jialiangensis
